# Time to imagine moving: Simulated motor activity affects time perception

**DOI:** 10.3758/s13423-021-02028-2

**Published:** 2021-12-16

**Authors:** Michiel M. Spapé, Ville J. Harjunen, Niklas Ravaja

**Affiliations:** Department of Psychology and Logopedics, Faculty of Medicine, University of Helsinki, Haartmaninkatu 3, 00290 Helsinki, Finland

**Keywords:** Time perception, Perception-action, Imagery, Motor control

## Abstract

Sensing the passage of time is important for countless daily tasks, yet time perception is easily influenced by perception, cognition, and emotion. Mechanistic accounts of time perception have traditionally regarded time perception as part of central cognition. Since proprioception, action execution, and sensorimotor contingencies also affect time perception, perception-action integration theories suggest motor processes are central to the experience of the passage of time. We investigated whether sensory information and motor activity may interactively affect the perception of the passage of time. Two prospective timing tasks involved timing a visual stimulus display conveying optical flow at increasing or decreasing velocity. While doing the timing tasks, participants were instructed to imagine themselves moving at increasing or decreasing speed, independently of the optical flow. In the direct-estimation task, the duration of the visual display was explicitly judged in seconds while in the motor-timing task, participants were asked to keep a constant pace of tapping. The direct-estimation task showed imagining accelerating movement resulted in relative overestimation of time, or time dilation, while decelerating movement elicited relative underestimation, or time compression. In the motor-timing task, imagined accelerating movement also accelerated tapping speed, replicating the time-dilation effect. The experiments show imagined movement affects time perception, suggesting a causal role of simulated motor activity. We argue that imagined movements and optical flow are integrated by temporal unfolding of sensorimotor contingencies. Consequently, as physical time is relative to spatial motion, so too is perception of time relative to imaginary motion.

## Introduction

Time is a fundamental physical dimension, yet its subjective experience is surprisingly flexible and affected by perception, cognition, and emotion. Thus, the perception of durations depends on rhythm (Treisman et al., 1990) and context (Nakajima et al., 2004; van Erp & Spapé, 2008). It is also affected by crossmodal perception, notably in interaction with spatial perception (Cohen et al., 1953; Suto, 1951). Central processes like memory and attention likewise determine time perception, as for example with infrequent, relevant, and novel “oddball” stimuli appearing to last longer (Ranganath & Rainer, 2003; Tse et al., 2004). Finally, affective states influence time perception: Pleasant experiences result in temporal compression (Gable & Poole, 2012), whereas arousal causes temporal dilation (Droit-Volet et al., 2011; Harjunen et al., 2021).

A central, time-keeping mechanism is commonly inferred to mechanistically explain our ability to estimate time. The principal model, scalar expectancy theory (Gibbon, 1977; Wearden & McShane, 1988) identifies two components: a pacemaker, which “ticks” at an unknown rate, and an accumulator, which polls the pacemaker if attention is focused on making a temporal judgement. As a central mechanism, the theory accounts for systematic timing errors as arising from either a change of the pacemaker’s rate, or due to attentional resource allocation to the timing task. Thus, it explains temporal dilation effects of threatening stimuli as an increased pace of the internal clock (Droit-Volet & Gil, 2009) and of oddballs as increased attention towards temporal processing (Tse et al., 2004).

While models such as the pacemaker-accumulator ascribe a central place in cognitive processing to timing, several observations suggest motor processes do affect temporal perception. Indeed, already in 1889, Hugo Münsterberg observed that during a time-reproduction task, participants involuntarily repeated their auxiliary motions, copying the respiratory actions made during intervals that were to be reproduced. This caused him to suggest we judge the passage of time by relying on the feelings resulting from muscular tension and relaxation (Münsterberg, 1889; see also note 32 in James, 1890). Thus, our sense of time may not merely rely on a central mechanism, as “late,” response selection, and execution-related processes play a role in temporal judgements.

Over the subsequent years, studies have repeatedly shown that motor activity and time perception are not only correlated, but interdependent. Thus, for example, O’Regan et al. (2017) observed an interrelationship between handedness, timed motor behaviour, and time experience. Interval timing is also a critical part of the experience of music, and while music perception is well known to affect time perception, it is now becoming clear that musical action and embodiment affect how we experience music in time (Maes et al., 2014). For example, a bisection task experiment showed temporal acuity increases as a consequence of voluntarily initiating auditory sequences (Iordanescu et al., 2013). However, perhaps the most famous experience of movement affecting time comes from the phenomenology of endurance sports practitioners, who, lost in the flow of the motion, may lose all sense of time (Csikszentmihalyi, 2000; Stoll, 2019). As with the time-perception literature in general, the causal role of action in flow experience remains unclear as research has predominantly focused on cognitive aspects, such as cognitive load and attention.

An interdependent relationship between motor activity and time perception follows from perception-action theories, such as the sensorimotor account of awareness (O’Regan & Noë, 2001), embodied cognition frameworks (Wilson, 2002), and common coding theory (Prinz, 1990). Thus, while the sensorimotor account views conscious perception as knowing how movements result in sensory consequences, we propose that awareness of time derives from knowing how movements result in sensory consequences over time. Embodied cognition theory explains time perception as the experience of the body (Wittmann et al., 2010), and the body’s action capabilities have indeed been found to affect time perception (Chambon et al., 2008; Thelen, 1995). Finally, from common coding theory (Hommel et al., 2001; Prinz, 1990), which argues for a representational equivalence between perception and action, it follows that performing musical sequences increases temporal acuity due to the cross-modal action effects coupling (Maes et al., 2014). Whether due to high-level embodied cognition or due to the natural, rhythmic sequences of the musculoskeletal system itself (Todd et al., 1999), cognitive neuroscience suggests the same neural structures are involved both in temporal planning and in movement coordination (Schubotz et al., 2000).

Movement may therefore affect time perception, yet in an everyday scenario, such as during a running exercise, two potential causes are typically confounded during real-world performance (Matthews & Meck, 2014). First, natural movement commonly involves optical flow, the pattern of velocity of a scene relative to the observer (Gibson, 1950), and the mere perception of speed within visual patterns causes time dilation (Kanai et al., 2006). Second, physical exertion naturally leads to arousal, and although the endorphin model of “runner’s high” lost its academic cachet (Stoll, 2019), arousal does affect temporal perception (Droit-Volet & Gil, 2009). Thus, to investigate how motor activity itself affects time perception requires controlling for these normally covarying factors.

To investigate how motor activity affects time perception, the present study used a motor imagery procedure. According to the motor-simulation theory, imagined actions involve the same cognitive representations and neural substrates involved in executing these actions (Jeannerod, 2001). This explains the strong similarities between imagery and execution, such as the time it takes to imagine walking to a target being strongly related to the actual walking time (Decety et al., 1989). Imagined locomotion also conforms to Fitts' Law (Fitts, 1954), its duration related to both the distance of the target and the difficulty of reaching it (Stevens, 2005). Likewise, manipulating the physical walking speed using a treadmill was found to cause the imagined speed to recalibrate (Kunz et al., 2009). The present aim, however, concerns not the timing of an action, but rather the effect of action imagery on time perception itself.

Specifically, the present experiment used motor imagery, assuming this causes subliminal motor activity, and predicting increased movement to result in perceived time to speed up relative to imagining decreased movement. To test the hypothesis, participants were requested to imagine running faster and faster or walking slower and slower, while timing the duration of videos of a starfield moving in either increasing or decreasing speed.

Two common prospective time-perception tasks were used to test subjective time. In the explicit, *time-estimation* task, participants were asked to keep track of time by estimating the duration of the video while performing the movement-imagining task. In this variant of the common verbal estimation task (Zakay, 1993), participants were instructed to explicitly count ”seconds,” which has been suggested to dramatically improve estimates (Grondin et al., 1999; Killeen & Weiss, 1987). In the implicit, *motor-timing* task, participants likewise performed the movement-imagining task, but were now requested to maintain a steady tapping pace throughout the course of the videos. Tapping tasks have a long history in the temporal cognition literature, which related them to other paradigms (Cahoon, 1969). More recent work argues motor-timing variability is dissociable from interval estimation (Robertson et al., 1999), while tapping speed drift is related to the central timekeeper pace (Repp, 2005).

We therefore preregistered (https://aspredicted.org/v7nm5.pdf) the following predictions for both tasks. First, if the star field’s acceleration or deceleration were to affect time perception, this would show visual motion speed affects subjective time. Second, if the mental imagination were to affect time perception, this would show motor simulation affects subjective time.

## Methods

### Participants

Participants were recruited from mailing lists to take part in an online experiment on time-perception. Following expression of interest, they received instructions on the task and were informed of their rights – including the right to cancel their participation at any time without fear of any consequences – via email. They signed the informed consent by clicking on the included E-Prime Go link. The average age of the 35 participants (see pre-registration on power considerations) who agreed to participate was 26.7 years (SD = 6.1), and 23 identified as female, ten as male, and two as non-binary.

### Stimuli and apparatus

The experiment made use of 3-min optical flow videos in which a starfield was shown as approaching towards the camera. Adobe premiere was used to create two versions of the video, using the optical flow algorithm to adjust the speed. For the decelerating conditions, the speed was adjusted to go 2.5x slower after 3 s and 2.5x slower (i.e., 6.25x slower than initial) after 6 s. For the accelerating conditions, the speed was increased by 2x after the same intervals (i.e., the second 4x faster than initial). Thereafter, the videos were trimmed down to 20 s, any further running time adjustments being done at experimental runtime.

The experiment was designed using E-Prime 3.0.3.80, compiled using E-Prime Go 1.0.2.41 to run locally on participants’ home computers. For the majority, this meant that 32 participants ran at a refresh rate of 60 Hz (SD = 0.09 Hz), and the remainder at 40, 65, and 75 Hz, as estimated by E-Prime. Almost all participants used Windows 10 (Windows being a requirement for using E-Prime Go), although one used Windows 7 and another 8.1. Most participants used a display with a resolution of 1,920 x 1,080 (n = 22) or 1,366 x 768 (n = 8), while resolutions in between these two were uncommon (n = 2), as were higher resolution displays (n = 3).

### Procedure

The experiments involved two separate tasks: a time-estimation task and a motor-timing task. These were presented in four blocks, their order counter-balanced as either 1-2-1-2 or 2-1-2-1.

**Fig. 1:**
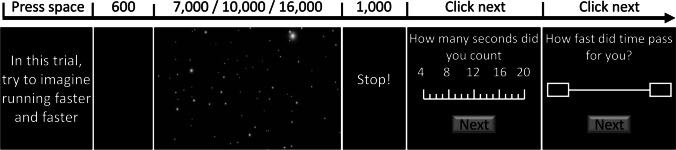
Time-estimation task. Following a blank screen, participants timed the duration of a star field, which was displayed for 6, 10, or 16 s, while imagining they were running, walking, or neither. Subjective time and passage of time were reported on separate scales

The *time-estimation task* was a prospective timing task, in which participants were instructed to estimate the duration of the videos by mentally counting seconds as they elapsed. In each trial, an instruction was displayed, asking participants to either “imagine walking slower and slower,” “imagine running faster and faster,” or “watch passively.” After pressing the space key, a 600-ms black screen was shown before the videos were presented for a duration of 7, 10, or 16 s (see Fig. 1). Following a 1,000-ms cue to stop counting, a scale from 4 to 20 s was displayed to indicate with the mouse the number of seconds they had counted. After they responded, participants were asked “how fast did time pass for you,” and to indicate their judgement of the passage of time on a 100-point visual analogue scale with “extremely slowly” and “extremely fast” at the endpoints (Kübel & Wittmann, 2020).

**Fig. 2:**
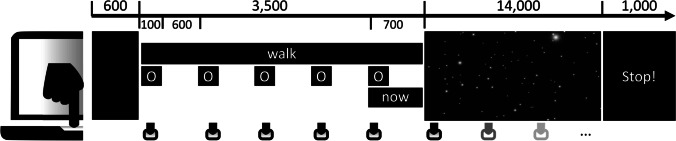
Motor-timing task. Participants tapped on the spacebar along with the circle cue while an instruction cue to imagine walking, running, or neither, was displayed. After the fourth beat, they were requested to maintain the tapping pace as the starfield videos were displayed

The *motor-timing task* used the same videos, but instead of mentally counting seconds, participants were requested to tap on the spacebar along with a steady pace of one beat per 700 ms. Each trial, they were instructed to “imagine walking slower and slower,” “imagine running faster and faster,” or “watch passively” while tapping. As shown in Fig. 2, following a 600-ms blank inter-trial interval, a black screen, a cue reminding of the task instruction (“walk,” “run,” or “just watch”) was presented for 3,500 ms one line above the center of the screen, while a cue (“O”) was intermittently shown with a stimulus-onset asynchrony of 700 ms and a duration of 100 ms to set the pace for the tapping, the last inter-stimulus interval additionally showing the word “now” as a cue for the video to start. Subsequently, the video was shown and participants were to maintain the even pace of tapping for 14,000 ms until the stop cue appeared.

### Design

The time-estimation task used a fully orthogonal experimental design with *presentation time* (7, 10, and 16 s), *optical flow* (fast and slow), and *imagery* (accelerating, decelerating, and neutral) as factors. *Imagery* was operationalized via instructing participants to imagine running faster and faster (accelerating), imagine walking slower and slower (decelerating), or not engage in imagery but watch passively (neutral). Each of the 18 combinations of factors was repeated four times across two blocks of 36 trials. The confirmatory part of the analysis used all three factors within a repeated-measures ANOVAs with estimated time (s) as dependent. We furthermore used the same analysis in the exploratory part of our analysis to determine whether *presentation time*, *optical flow*, and *imagery* also affected subjective passage of time.

The motor-timing task used a similar, orthogonal design, but with only optical flow and imagery condition as factors, and each of the six trial types repeated six times per block. To make tapping speed comparable across conditions, we estimated the inter-tap interval over the course of the trial, time-locked to the onset of the video by temporal interpolation of the tapping speed at constant intervals analogous to event-related cardiac activity analysis (Spapé et al., 2017). In this instance, the inter-tap interval between first and last response was interpolated to obtain continuous tapping speeds at a resolution of 10 Hz, while discarding inter-tap intervals < 100 ms and > 1,200 ms as artefactual. In the confirmatory part of the analysis, we used the same three-way ANOVA as with the time-estimation task, but for two changes. Firstly, instead of *presentation time*, *time period* was used to describe the analysis bins within trials of 0–4 s, 4–9 s, and 9–14 s, thus to determine whether any optical flow effect would arise in response to the star field’s first change in speed (at 3 s), its second (at 6 s), or at a later point. Secondly, the dependent variable was the averaged inter-tap interval (ITI) within these bins. An additional, exploratory part of the analysis used the entire trial, running sliding two-way repeated-measures ANOVAs for optical flow and imagery on each 100-ms bin.

For further details on the preprocessing of ITIs, please see the Open Science Framework project at osf.io/m69wy/, which also hosts the used stimuli, experimental code, and all data for this study.

## Results

Outliers were removed from analysis separately for the time-estimation and motor-timing task (https://aspredicted.org/v7nm5.pdf). One participant did not reliably distinguish between the three intervals (Z scores -0.29, 0.33) and was removed from the time-estimation analysis (remaining n = 34). In the motor-timing task, participants with highly unstable ITIs or ≤ six trials per design cell were removed (remaining n = 31).

### Time-estimation task

**Fig. 3:**
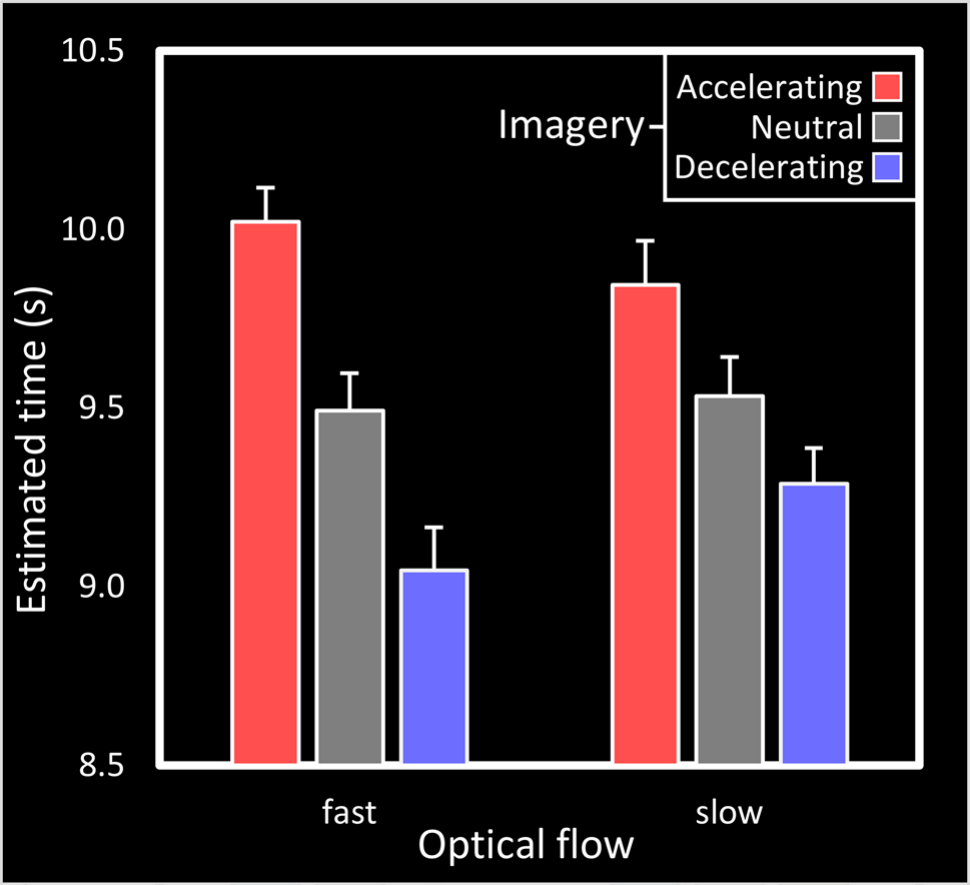
Effects of imagery and optical flow on estimated time. Grey (neutral) bars refer to the passive viewing condition, while accelerating and decelerating conditions to those in which participants were instructed to imagine running faster and faster or slower and slower, respectively. Error bars display within-participant standard errors

In the confirmatory part of the analysis, a repeated-measures ANOVA was conducted on the estimated time (s) with *presentation time* (7, 10, and 16 s), *optical flow* (fast, slow), and *imagery* (accelerating, decelerating, and neutral) as factors. This showed significant main effects of *presentation time*, F (1.11, 36.46) = 613.10, p < .0001, and *imagery*, F (1.62, 53.52) = 17.07, p < .0001, but not *optical flow*, F (1, 33) = 0.50, p = .49. Estimated times were generally underestimated, with estimations for 7, 10, and 16 s being 6.39 , 8.81, and 13.42 s. More interestingly, accelerating imagery increased time estimates relative to neutral (M = 0.42, SE = 0.12), while decelerating imagery decreased time estimates (M = -0.35, SE = 0.11). An interaction between *presentation time* and *imagery*, F (2.91, 96.14) = 6.26, p = .0007, indicated that this effect was enhanced at longer time intervals relative to shorter intervals (accelerating-decelerating at 7, 10, 16 s: 0.43, 0.73, 1.14). Finally, an interaction between *imagery* and *optical flow* was found, F (1.92, 63.23) = 6.50, p = .003. Fast optical flow enhanced the effect of *imagery*, with the difference between accelerating and decelerating imagery being larger in fast (mean difference, D = 0.97 s) than in slow optical flow conditions (D = 0.56 s, see Fig. 3).

An exploratory repeated-measures ANOVA on passage of time judgements was conducted in a manner analogous to the time estimations, but with response on the visual-analogue scale as dependent. *Presentation time*, F (1.09, 35.99) = 12.70, p = .0008, and *optical flow*, F (1, 33) = 26.80, p < .0001, significantly affected passage-of-time judgements, while *imagery* did not, F (1.57, 51.90) = 2.98, p = .07. Longer *presentation times* elicited slower passage-of-time responses (at 7, 10, 16 s: 53.5; 50.1; 16: 45.0), and fast *optical flow* prompted faster passage of time responses (53.3 vs. 45.8). *Presentation time* also interacted with *optical flow*, F (1.55, 51.00) = 3.67, p = .043, with the effect of *optical flow* being somewhat more pronounced over 16-s *presentation times* (mean difference D = 9.44) than 10 (D = 7.08) and 7 s (D = 5.94).

Finally, the three-way interaction between *presentation time*, *optical flow*, and *imagery* was also significant, F (3.05, 112.16) = 3.32, p = .022. Separate ANOVAs with *optical flow* and *imagery* as factors for each *presentation time* were conducted to explore this interaction. These showed a significant interaction for 16-s *presentation times*, F (1.59, 52.55) = 4.69, p = .020, with the effect of *optical flow* larger for accelerating (D = 11.99) and neutral *imagery* (D = 10.47) than for decelerating *imagery* (D = 5.86). No significant interaction was observed for shorter presentation times, *p*s > .17.

### Motor-timing task

**Fig. 4:**
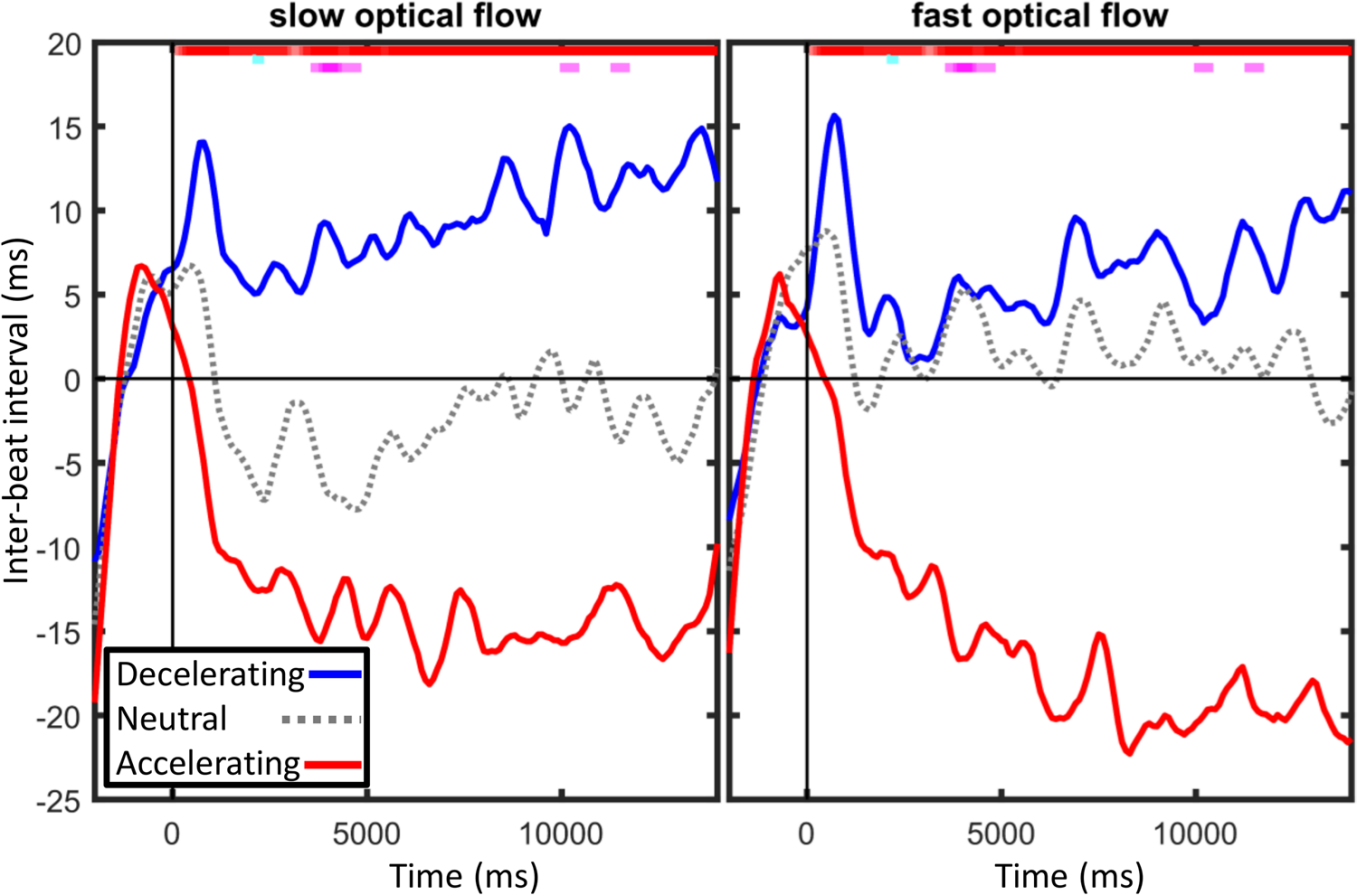
Averaged interpolated tapping speed as inter-beat interval, as affected by optical flow and imagery conditions. The top of each graph shows the outcome of sliding repeated-measures ANOVA on the average of each 100-ms bin. Red dots show main effects of imagery significant (p unadj. < .05), turquoise dots the main effect of optical speed, and pink dots the interaction between the two factors

In a confirmatory repeated-measures ANOVA on the average ITI during motor timing with *time period* (0–4 s, 4–9 s, 9–14 s), *optical flow*, and *imagery* as factors, neither *time period* nor *optical flow*, Fs < 1.31, ps > .26, significantly affected ITI, while *imagery* did, F (1.34, 40.23) = 11.58, p = .0006. Accelerating *imagery* decreased ITI (increased speed with 14.3 ms vs. baseline) relative to neutral (increased speed -0.2 ms) and decelerating *imagery* (decreased speed with 8.1 ms). *Time period* significantly interacted with *imagery*, F (1.90, 57.03) = 6.27, p = .004, with the effect of *imagery* increasing with the later bins, as can also be seen in Fig. 4.

A further exploratory analysis used a series of two-way repeated-measures ANOVAs for each 100-ms bin during the entire trial. The outcome, presented in Fig. 4, shows an initial deceleration of tapping prior to the video onset (time 0), followed by a strong effect of *imagery* from ca. 300 ms onwards. That is, accelerating *imagery* generally increased tapping speed while decelerating *imagery* decreased it. While the number of tests (140) precludes meaningful statistical inference, the effect of acceleration appeared more pronounced in fast optical flow and the effect of deceleration in slow optical flow.

## Discussion

Two prospective timing tasks measured the effects of optical flow and movement imagination on subjective time. The explicit, time-estimation task showed accelerating imagined movement to strongly increase perceived duration. Combining the speeding up during imagined movement with accelerating optical flow increased this effect, suggesting perception-action coupling affects temporal perception. Interestingly, the experience of the passage of time, as judged through self-reports, was affected by optical flow, but not motor imagery, confirming a dissociation between time estimations and passage-of-time judgements (Wearden, 2015).

The implicit, motor-timing task replicated the main findings from the time estimation task. In terms of the first preregistered prediction, the star field’s acceleration did not affect time perception, suggesting visual motion speed does not affect subjective time. However, in terms of the second prediction, the mental imagination did affect time perception, indicating that motor simulation does affect subjective time. Contrary to the findings from the time-estimation task, however, no clear interaction between imagery and visual flow was found, although the exploratory analysis here suggests the interaction may merely be attenuated and not removed altogether.

While the study provides evidence that motor imagery affects time perception, we would not go so far as to suggest perceptual and central processes do not play any role. Indeed, the present study did not replicate previously observed effects of optical flow on time estimation (Kanai et al., 2006) and reproduction (Verde et al., 2019). However, these previous studies measured time perception at the short-range interval, while the here-reported experiments used intervals between 7 and 16 s. Theories have long argued interval timing between 0 and 3 s differs from longer-range estimates (Münsterberg, 1889; Penney & Vaitilingam, 2008; Poppel, 2004), resulting in functional (Grondin, 2010) and neural dissociations (Wittmann, 2014). Furthermore, variable Weber fractions in time perception (Grondin, 2001) may mean a small effect of optical flow to not be readily in long-interval time estimation.

Imagining movement, however, had a clear effect on time perception. We argued in line with sensorimotor theory that our understanding of the passage of time need not only derive from sensory markers – so-called *Zeitgebers* (Pittendrigh, 1981; Sharma & Chandrashekaran, 2005) *–* but could be informed through the motor system and perception of actions. Motor imagery, commonly understood to involve simulated motor activity, was indeed shown to affect time perception. Furthermore, when the accelerating motor simulation was combined with congruent visual optical flow, the effect was most pronounced. This suggests that the effect relied on a coupling of perception and action: Imagining oneself as seeing the consequences of moving.

However, an alternative perspective on the results from the pacemaker-accumulator model would be that the increased speed of tapping and the temporal overestimation indicate a common mechanism: perhaps simulated running causes arousal? Indeed, arousal has been found to cause temporal dilation (Gil & Droit-Volet, 2012; Özoğlu & Thomaschke, 2021). This interpretation, or one involving the motor imagery to require attentional resources, cannot account for opposite effects of accelerating and decelerating imagery relative to the neutral condition. That is, a relative temporal underestimation was observed for walking in the decelerating condition compared to passive watching, while simulated walking should still involve both arousal and attentional resources.

A more fitting alternative interpretation involves the so-called *kappa effect*, which refers to the observation that the temporal interval between two markers is affected by their spatial distance (Kuroda et al., 2016; Price-Williams, 1954; Yoblick & Salvendy, 1970). Yet this explanation implies a sensorimotor or embodied framework in action. That is, no true spatial difference was presented within the stimulus, any more than watching a science-fiction movie causes virtual locomotion; indeed, optical flow had little effect. The effect instead relied on imagining oneself moving within the star field. Therefore, one might understand the pattern of results as a motor or *ideomotor* kappa effect. Indeed, temporal overestimation was maximal when the imagined movement corresponded with optical flow, giving the impression that one’s imagined motor activity resulted in locomotion. In this case, the increased distance travelled in mental locomotion may have given rise to changes in perceived time, analogous to the effects of imagination on action timing (Decety et al., 1989; Stevens, 2005).

Yet, optical flow did not increase the effect of mental imagery in the implicit, tapping task, which suggests that action execution here hindered the sensorimotor effect. Note, however, that tapping is no mere indicator of timing, but contributes a rhythmic, sensorimotor aspect to the task. This may have resulted in motor interference disrupting the visuomotor integration between imagined movement and optical flow (cf. Stevens, 2005). Alternatively, the mental imagery in the motor-timing task might have been coupled to the haptic consequences of tapping, discounting visual motion. Either interpretation underlines the importance of action stages as critical to time perception, rather than presenting a pure metric for studying its central mechanism.

Finally, the tasks used to estimate the perception of time place important limitations on the generalizability of the results. While the two tasks depend on timing mechanisms in a manner similar to daily-life operations, they are less informative on the experience of time. While the findings from the passage-of-time judgments point to a potential role of imagery at longer durations, further investigation needs to determine whether mental imagery affects other aspects of time perception. If the temporal replication and discrimination tasks rely on the same mechanism as verbal estimation, as has been observed for the former (Robertson et al., 1999), a pattern of results similar to the explicit counting task should be observed. Furthermore, if imagery affects time experience, we would expect anticipation tasks to be similarly affected, which should be confirmed via a variable foreperiod task (Grondin & Rammsayer, 2003) or EEG measurement (Walter et al., 1964).

In sum, this study showed perception of time is more than the accumulation of sensory information and involves motor activity and perception/action integration. As a result, our understanding of time goes beyond obtaining a fleeting glimpse of the world as it goes by but is affected by how we interact with the environments and can thus inform how our intentions may unfold over time. Thus, as physical time is relative to spatial motion, so too is psychological time relative to imaginary motion.

## References

[CR1] Cahoon RL (1969). Physiological arousal and time estimation. Perceptual and Motor Skills.

[CR2] Chambon M, Droit-Volet S, Niedenthal PM (2008). The effect of embodying the elderly on time perception. Journal of Experimental Social Psychology.

[CR3] Cohen J, Hansel CEM, Sylvester JD (1953). A new phenomenon in time judgment. Nature.

[CR4] Csikszentmihalyi M (2000). *Beyond boredom and anxiety*.

[CR5] Decety J, Jeannerod M, Prablanc C (1989). The timing of mentally represented actions. Behavioural Brain Research.

[CR6] Droit-Volet S, Gil S (2009). The time–emotion paradox. Philosophical Transactions of the Royal Society B: Biological Sciences.

[CR7] Droit-Volet S, Fayolle SL, Gil S (2011). Emotion and time perception: Effects of film-induced mood. Frontiers in Integrative Neuroscience.

[CR8] Fitts PM (1954). The information capacity of the human motor system in controlling the amplitude of movement. Journal of Experimental Psychology.

[CR9] Gable PA, Poole BD (2012). Time flies when you’re having approach-motivated fun: Effects of motivational intensity on time perception. Psychological Science.

[CR10] Gibbon J (1977). Scalar expectancy theory and Weber’s law in animal timing. Psychological Review.

[CR11] Gibson, J. J. (1950). *The perception of the visual world. *Houghton Mifflin, Boston: MA.

[CR12] Gil S, Droit-Volet S (2012). Emotional time distortions: The fundamental role of arousal. Cognition & Emotion.

[CR13] Grondin S (2001). A temporal account of the limited processing capacity. Behavioral and Brain Sciences.

[CR14] Grondin S (2010). Timing and time perception: A review of recent behavioral and neuroscience findings and theoretical directions. Attention, Perception, & Psychophysics.

[CR15] Grondin S, Rammsayer T (2003). Variable foreperiods and temporal discrimination. The Quarterly Journal of Experimental Psychology Section A.

[CR16] Grondin S, Meilleur-Wells G, Lachance R (1999). When to start explicit counting in a time-intervals discrimination task: A critical point in the timing process of humans. Journal of Experimental Psychology: Human Perception and Performance.

[CR17] Harjunen, V. J., Spapé, M., & Ravaja, N. (2021). Anticipation of aversive visual stimuli lengthens perceived temporal duration. *Psychological Research*. 10.1007/s00426-021-01559-6 10.1007/s00426-021-01559-6 PMC9090676 34357421

[CR18] Hommel B, Müsseler J, Aschersleben G, Prinz W (2001). The Theory of Event Coding (TEC): A framework for perception and action planning. The Behavioral and Brain Sciences.

[CR19] Iordanescu L, Grabowecky M, Suzuki S (2013). Action enhances auditory but not visual temporal sensitivity. Psychonomic Bulletin & Review.

[CR20] James W (1890). *The Principles of Psychology: Vol*.

[CR21] Jeannerod M (2001). Neural simulation of action: A unifying mechanism for motor cognition. Neuroimage.

[CR22] Kanai R, Paffen CL, Hogendoorn H, Verstraten FA (2006). Time dilation in dynamic visual display. Journal of Vision.

[CR23] Killeen PR, Weiss NA (1987). Optimal timing and the Weber function. Psychological Review.

[CR24] Kübel SL, Wittmann M (2020). A German Validation of Four Questionnaires Crucial to the Study of Time Perception: BPS, CFC-14, SAQ, and MQT. International Journal of Environmental Research and Public Health.

[CR25] Kunz BR, Creem-Regehr SH, Thompson WB (2009). Evidence for motor simulation in imagined locomotion. Journal of Experimental Psychology: Human Perception and Performance.

[CR26] Kuroda T, Grondin S, Miyazaki M, Ogata K, Tobimatsu S (2016). The kappa effect with only two visual markers. Multisensory Research.

[CR27] Maes P-J, Leman M, Palmer C, Wanderley M (2014). Action-based effects on music perception. Frontiers in Psychology.

[CR28] Matthews WJ, Meck WH (2014). Time perception: The bad news and the good. Wiley Interdisciplinary Reviews: Cognitive Science.

[CR29] Münsterberg H (1889). *Beiträge zur Experimentellen Psychologie*.

[CR30] Nakajima Y, Ten Hoopen G, Sasaki T, Yamamoto K, Kadota M, Simons M, Suetomi D (2004). Time-shrinking: The process of unilateral temporal assimilation. Perception.

[CR31] O’Regan JK, Noë A (2001). A sensorimotor account of vision and visual consciousness. Behavioral and Brain Sciences.

[CR32] O’Regan L, Spapé MM, Serrien DJ (2017). Motor timing and covariation with time perception: Investigating the role of handedness. Frontiers in Behavioral Neuroscience.

[CR33] Özoğlu E, Thomaschke R (2021). Early posterior negativity indicates time dilation by arousal. Experimental Brain Research.

[CR34] Penney, T. B., & Vaitilingam, L. (2008). Imaging time. *Psychology of Time*, 261–294.

[CR35] Pittendrigh CS (1981). Circadian systems: Entrainment. In *Biological rhythms*.

[CR36] Poppel E (2004). Lost in time: A historical frame, elementary processing units and the 3-second window. Acta Neurobiologiae Experimentalis.

[CR37] Price-Williams DR (1954). The kappa effect. Nature.

[CR38] Prinz, W. (1990). A common coding approach to perception and action. In: *Relationships between perception and action* (pp. 167–201). Springer.

[CR39] Ranganath C, Rainer G (2003). Neural mechanisms for detecting and remembering novel events. Nature Reviews Neuroscience.

[CR40] Repp BH (2005). Sensorimotor synchronization: A review of the tapping literature. Psychonomic Bulletin & Review.

[CR41] Robertson SD, Zelaznik HN, Lantero DA, Bojczyk KG, Spencer RM, Doffin JG, Schneidt T (1999). Correlations for timing consistency among tapping and drawing tasks: Evidence against a single timing process for motor control. Journal of Experimental Psychology: Human Perception and Performance.

[CR42] Schubotz RI, Friederici AD, Von Cramon DY (2000). Time perception and motor timing: A common cortical and subcortical basis revealed by fMRI. Neuroimage.

[CR43] Sharma, V. K., & Chandrashekaran, M. K. (2005). Zeitgebers (time cues) for biological clocks. *Current Science*, 1136–1146.

[CR44] Spapé MM, Harjunen V, Ravaja N (2017). Effects of touch on emotional face processing: A study of event-related potentials, facial EMG and cardiac activity. Biological Psychology.

[CR45] Stevens JA (2005). Interference effects demonstrate distinct roles for visual and motor imagery during the mental representation of human action. Cognition.

[CR46] Stoll, O. (2019). Peak performance, the runner’s high, and flow*.* In M. H. Anshel, S. J. Petruzzello, & E. E. Labbé (Eds.), *APA handbook of sport and exercise psychology, Vol. 2. Exercise psychology* (pp. 447–465). American Psychological Association. 10.1037/0000124-023

[CR47] Suto Y (1951). The Effect of Space on Time Estimation (S-ffect) in Tactual Space (I). Japanese Journal of Psychology.

[CR48] Thelen, E. (1995). Time-scale dynamics and the development of an embodied cognition. In R. F. Port & T. van Gelder (Eds.), *Mind as motion: Explorations in the dynamics of cognition* (pp. 69–100). The MIT Press.

[CR49] Todd NPM, O’Boyle DJ, Lee CS (1999). A sensory-motor theory of rhythm, time perception and beat induction. Journal of New Music Research.

[CR50] Treisman M, Faulkner A, Naish PL, Brogan D (1990). The internal clock: Evidence for a temporal oscillator underlying time perception with some estimates of its characteristic frequency. Perception.

[CR51] Tse PU, Intriligator J, Rivest J, Cavanagh P (2004). Attention and the subjective expansion of time. Perception & Psychophysics.

[CR52] van Erp, J. B., & Spapé, M. M. (2008). Time-shrinking and the design of tactons. In: *International Conference on Human Haptic Sensing and Touch Enabled Computer Applications* (pp. 289–294). Berlin, Heidelberg: Springer.

[CR53] Verde LL, Alais D, Burr DC, Morrone MC, MacDougall H, Verstraten FA (2019). Time dilation effect in an active observer and virtual environment requires apparent motion: No dilation for retinal-or world-motion alone. Journal of Vision.

[CR54] Walter WG, Cooper R, Aldridge VJ, McCallum WC, Winter AL (1964). Contingent Negative Variation: An Electric Sign of Sensori-Motor Association and Expectancy in the Human Brain. Nature.

[CR55] Wearden JH (2015). Passage of time judgements. Consciousness and Cognition.

[CR56] Wearden JH, McShane B (1988). Interval production as an analogue of the peak procedure: Evidence for similarity of human and animal timing processes. The Quarterly Journal of Experimental Psychology.

[CR57] Wilson M (2002). Six views of embodied cognition. Psychonomic Bulletin & Review.

[CR58] Wittmann, M. (2014). *Embodied time: The experience of time, the body, and the self. *In Arstila V, Lloyd D (Eds.), *Subjective time: The philosophy, psychology, and neuroscience of temporality *(pp. 507–523). Cambridge, MA: MIT press.

[CR59] Wittmann M, Simmons AN, Aron JL, Paulus MP (2010). Accumulation of neural activity in the posterior insula encodes the passage of time. Neuropsychologia.

[CR60] Yoblick DA, Salvendy G (1970). Influence of frequency on the estimation of time for auditory, visual, and tactile modalities: The kappa effect. Journal of Experimental Psychology.

[CR61] Zakay D (1993). Time estimation methods—Do they influence prospective duration estimates?. Perception.

